# The signaling pathways of traditional Chinese medicine in treating diabetic retinopathy

**DOI:** 10.3389/fphar.2023.1165649

**Published:** 2023-06-19

**Authors:** Wencan Li, Qichang Xing, Zheng Liu, Renzhu Liu, Yixiang Hu, Qingzi Yan, Xiang Liu, Jiani Zhang

**Affiliations:** ^1^ Department of Clinical Pharmacy, Xiangtan Central Hospital, Xiangtan, Hunan, China; ^2^ Department of Pharmacy, Xiangtan Central Hospital, Xiangtan, Hunan, China

**Keywords:** diabetic retinopathy, traditional Chinese medicine, signaling pathway, mechanism, review

## Abstract

Diabetic retinopathy (DR) is one of the common diabetic microvascular complications that occurs in the eyes and is closely associated with vision loss in working adults. However, the clinical treatment of DR is limited or accompanied by a large number of complications. Therefore, the development of new drugs for the treatment of DR is urgently needed. Traditional Chinese medicine (TCM) is widely used to treat DR in China, and its multi-pathway and multi-level characteristics can effectively address the complex pathogenesis of DR. Growing evidence suggests that inflammation, angiogenesis, and oxidative stress are the core pathological mechanisms in the development of DR. This study innovatively considers the aforementioned processes as the fundamental unit and sheds light on the molecular mechanisms and potential of TCM against DR in terms of signaling pathways. The results showed that NF-κB, MAPK/NF-κB, TLR4/NF-κB, VEGF/VEGFR2, HIF-1α/VEGF, STAT3, and Nrf2/HO-1 are the key signaling pathways for the treatment of DR by TCMs, which involved curcumolide, erianin, quercetin, blueberry anthocyanins, puerarin, arjunolic acid, ethanol extract of *Scutellaria barbata* D. Don, *Celosia argentea* L. extract, ethanol extract of *Dendrobium chrysotoxum* Lindl., Shengpuhuang-tang, and LuoTong formula. The purpose of this review is to update and summarize the signaling pathways of TCM in the treatment of DR and provide ideas for the development of new drugs against DR in the future.

## 1 Introduction

Diabetes mellitus (DM) is a chronic progressive disease characterized by persistent hyperglycemia. According to statistics from the International Diabetes Federation, there are currently 537 million adults having diabetes worldwide, and this number will continue to grow over time, with 800 million people expected by 2045. The ultimate outcome of DM is chronic diabetes complications, especially vascular complications, which are mainly divided into microvascular complications (retina, kidney, and nervous system) and macrovascular complications (cerebrovascular, cardiovascular, and peripheral vascular). These complications are a major cause of increased mortality and associated costs among diabetics and have become a global public health problem (Di et al., 2022; Wei et al., 2022; Yang and Yang, 2022). Diabetic retinopathy (DR) is one of the common diabetic microvascular complications that occurs in the eyes and is closely associated with vision loss in working adults and seriously reduces the quality of life. There are reported to be 103 million DR patients by 2022, and this number is expected to increase to 160 million by 2045 (Antonetti et al., 2021; Di et al., 2022; Pitale and Gorbatyuk, 2022).

**FIGURE 1:**
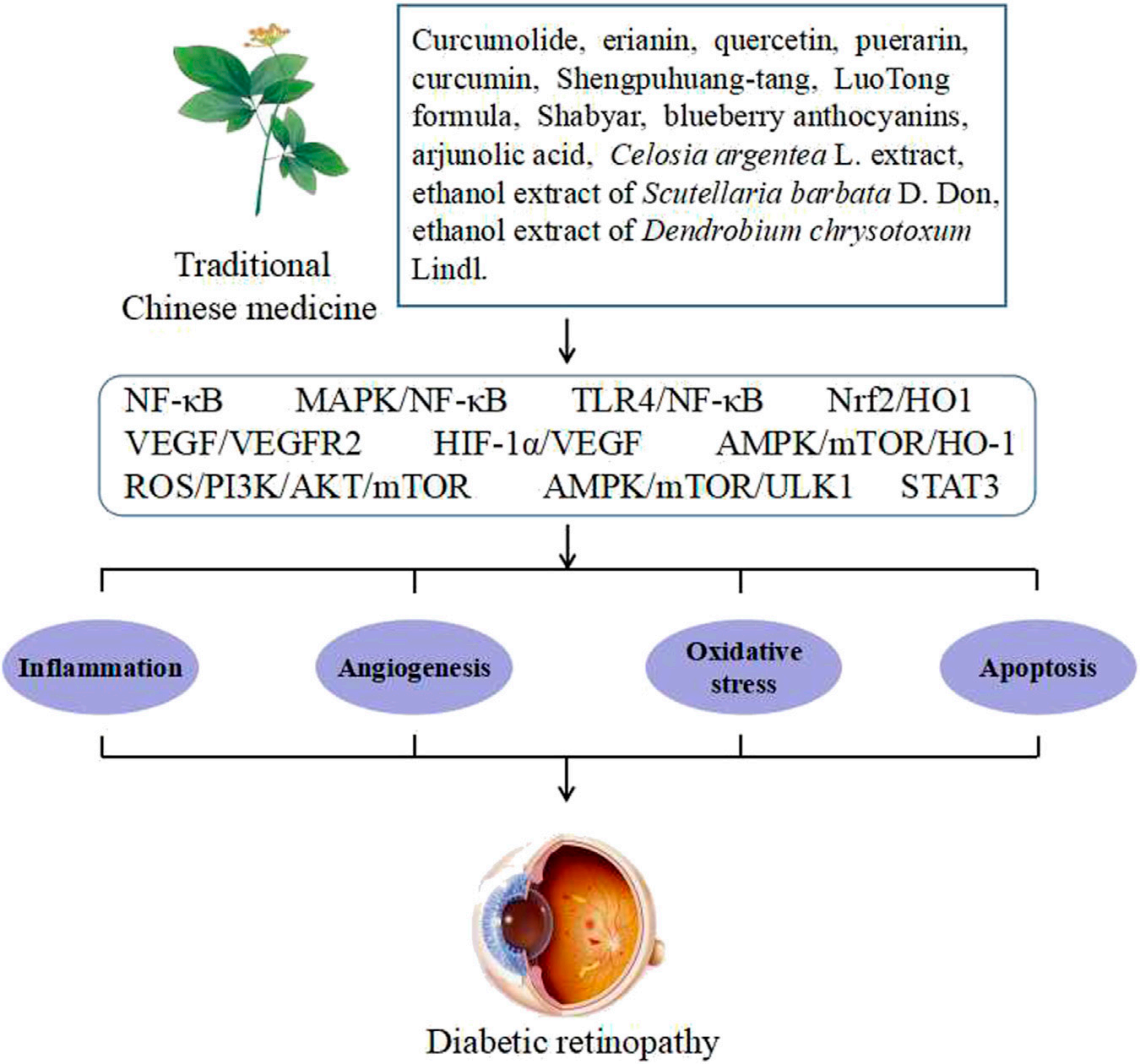
Potential signaling pathways of TCM for preventing and treating DR.

According to different pathological characteristics, DR can be divided into two stages: non-proliferative DR (NPDR) and proliferative DR (PDR). The main clinical manifestations of NPDR are microaneurysms, hemorrhage, and hard exudations, and the appearance of neovascularization indicates that the disease has entered the PDR stage. In addition, it is usually accompanied by retinal hemorrhage and detachment (Choo et al., 2021; Tomita et al., 2021). Owing to the concealment of the early onset of DR, it is easy to cause DR to be in the PDR stage by the time it is detected, by which time many retinal injuries cannot be reversed. Currently, the clinical treatment of DR is limited or is accompanied by a significant number of complications. For example, surgery and laser treatment may result in visual impairment or diabetic macular edema. Moreover, treatment with mainstream anti-vascular endothelial growth factor (VEGF) drugs may result in intraocular inflammation, infection, and rhegmatogenous retinal detachment. Therefore, the development of new drugs for the treatment of DR is urgently needed (Song et al., 2019; Sun et al., 2021). Traditional Chinese medicine (TCM) has been used in China for thousands of years. Over the past few years, many experimental studies have been conducted on TCM, which has demonstrated promising activity against DR. TCM is characterized by multiple components that act simultaneously on multiple targets in a synergistic manner to produce beneficial effects on the disease. The pathogenesis of DR is very complex, but after a long study period, scholars believe that the occurrence and development of DR are mainly related to inflammation, angiogenesis, oxidative stress, and apoptosis (Li W. C. et al., 2022). Currently, there is a lack of system reviews on the signaling pathways of TCM against DR. This review summarizes the signaling pathways of TCM against DR in terms of inflammation, angiogenesis, and oxidative stress, with the aim of providing ideas for developing new drugs against DR. Potential signaling pathways of TCM for preventing and treating DR are shown in Figure 1.

## 2 Signaling pathways in TCM intervening with inflammation of DR

**FIGURE 2:**
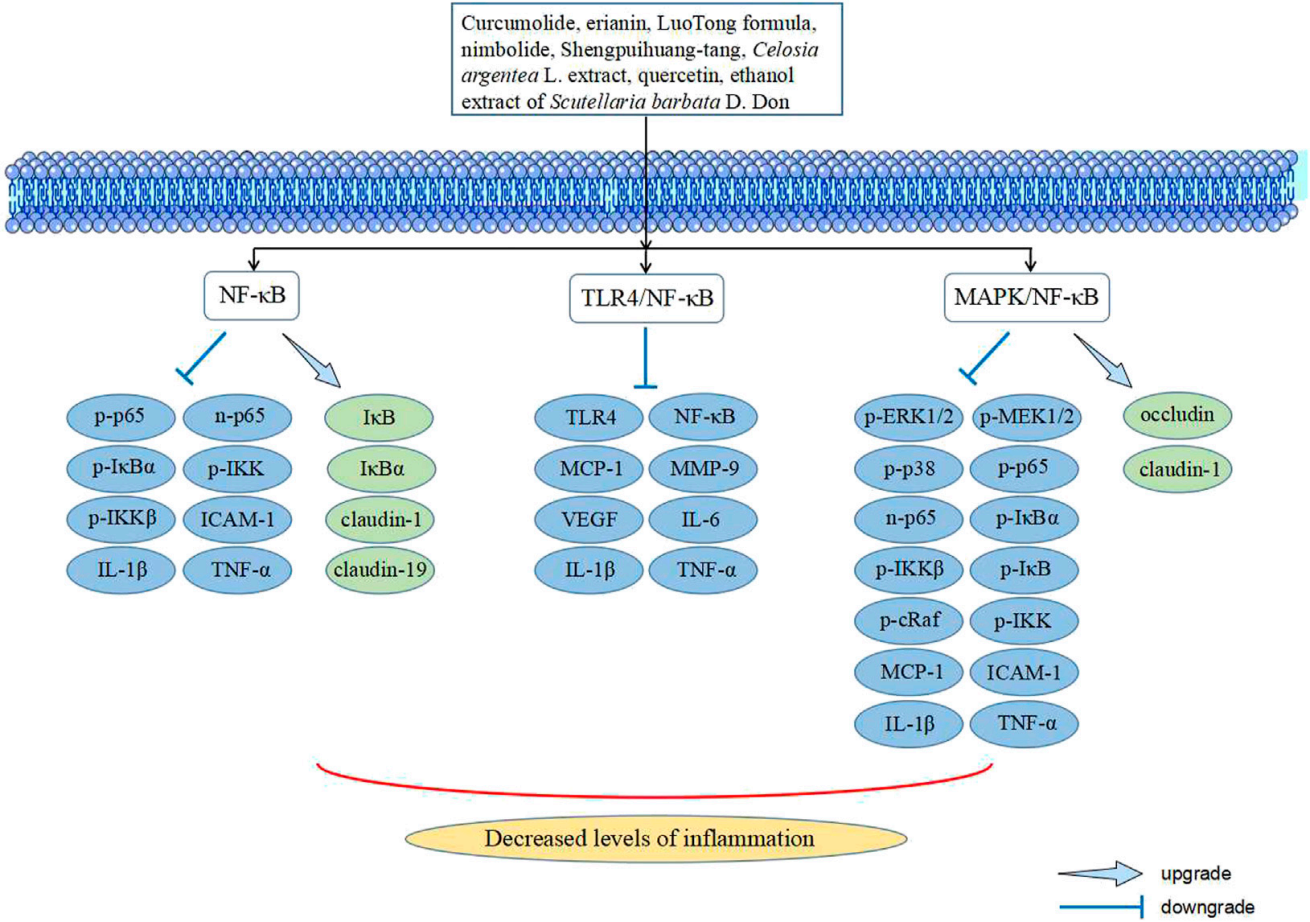
Signaling pathways in TCM intervening with inflammation of DR.

DR is a chronic low-inflammatory microvascular complication of diabetes, in which inflammation plays an important role at various stages of development. Persistent hyperglycemia is an important factor that causes retinal vascular inflammation, which leads to a series of changes in the structure and function of retinal blood vessel walls, such as increased vascular permeability, leukostasis, vascular leakage, and blood–retinal barrier (BRB) breakdown. Among them, leukocyte adhesion causes retinal vascular endothelial cell death, BRB breakdown, and increased vascular permeability. Increased vascular permeability is an important factor that causes vascular leakage, macular edema, and visual loss (Ai et al., 2020; Li W. C. et al., 2020; Carpi-Santos et al., 2022). In addition, the expression levels of various inflammatory mediators such as tumor necrosis factor-α (TNF-α), interleukin-1 (IL-1), and intercellular cell adhesion molecule-1 (ICAM-1) are increased under the stimulation of persistent hyperglycemia, which has been demonstrated in the vitreous cavity and serum of diabetic patients and various types of diabetic animal models. The anomalies of the aforementioned indicators usually do not appear alone because they influence each other on the basis of their own development; for example, TNF-α aggravates leukocyte adhesion and BRB breakdown, and ICAM-1 is closely related to leukocyte adhesion (Li W. C. et al., 2020; Khazeei Tabari et al., 2022; Kovoor et al., 2022). It has been reported that many TCMs exhibit against retinal inflammation in diabetes (Figure 2), and the signaling pathways involved are explained in the following paragraphs.

### 2.1 NF-κB signaling pathway

The NF-κB signaling pathway includes both classical and non-classical signaling pathways. The main difference is that the signaling pathway is activated differently. The classical NF-κB signaling pathway plays an important role in a variety of physiological and pathological processes, such as inflammation, oxidative stress, immunity, and apoptosis. The nuclear factor kappa B (NF-κB) family comprises five members: p50, p52, p65, c-Rel, and RelB. In general, the aforementioned members form a complex with the inhibitor of NF-κB (IκB) in the form of dimers because IκB can mask its nuclear location signal and cause NF-κB to exist in the cytoplasm in a non-activated form. When cells are stimulated, these signals are transmitted and activated by the IκB kinase (IKK) complex, resulting in the phosphorylation and ubiquitination of the IκB protein. IκB is then degraded and releases a dimer of NF-κB, which translocates to the nucleus to bind to DNA and promote transcription. Among them, the main components of the IKK complex are IKKα, IKKβ, and NEMO. IKKβ plays an important role in the inflammatory response. NF-κB is not activated when inflammatory factors (such as TNF-α and IL-1) stimulate IKKβ-deficient cells. Iκbα is a good substrate for IKK. It can bind to the most common heterodimer p65:p50 of NF-κB to prevent it from entering the nucleus and binding with DNA, which is the most classical member of the IκB protein family. In addition, IκBα is the strongest negative feedback factor in NF-κB activation, which ensures the rapid occurrence and completion of NF-κB activation. In the absence of IκBα, termination of the activated NF-κB signaling pathway is significantly delayed (Wang et al., 2019; Li W. C. et al., 2020; Wu et al., 2021).

Shengpuihuang-tang (ST) is composed of 11 types of TCM and has the effect of promoting blood circulation and removing blood stasis. It is often used in the treatment of DR in clinical practice, particularly NPDR (Du, 2018; Li W. C. et al., 2020). Li W. C. et al. (2020) found that ST alleviated vascular leakage and leukostasis, and downregulated the levels of inflammatory mediators TNF-α and ICAM-1 in the retinas of diabetic rats. In addition, further mechanistic research has shown that ST inhibited the degradation of IκBα, the nuclear transfer of p65, and the protein expression of p-p65 and p-IKKβ. These results suggested that ST may play a role in improving DR by inhibiting the NF-κB signaling pathway to reduce retinal inflammation. Guo et al. (2016) found that after *Celosia argentea* L. extract (CAE) intervention, the expression of the pro-inflammatory factors TNF-α, IL-1β, and IL-6 in the serum of diabetic mice was significantly decreased. In addition, albumin leakage and expression levels of VEGF, IκB, p-NF-κB, and p-IKK in retinal tissues were reversed by CAE. It is suggested that CAE may improve retinal inflammation in DR by inhibiting the activation of the NF-κB signaling pathway.

Moreover, ginger extract containing 5% 6-gingerol (Dongare et al., 2016), ethanol extract of *Scutellaria barbata* D. Don (Mei et al., 2017), polysaccharides of *Dendrobium candidum* (Li et al., 2016), catalpol (Wu and Du, 2021), and curcumolide (Dong et al., 2015) have shown significant effects in alleviating retinal inflammation in diabetes through a mechanism related to the inhibition of the NF-κB signaling pathway.

### 2.2 MAPK/NF-κB signaling pathway

The mitogen-activated protein kinase (MAPK) signaling pathway is a widespread class of silk/threonine protein kinases in eukaryotic cells that can be activated by physical stress, inflammatory cytokines, bacterial complexes, and other extracellular signals. MAPK is mainly composed of extracellular signal-regulated kinase 1/2 (ERK1/2), p38 MAPK, Jun N-terminal kinase (JNK), ERK5, ERK3/4, Nemo-like kinases, and ERK7. Among them, the first four are canonical MAPKs, and the last three are atypical MAPKs (Gao et al., 2014; Asl et al., 2021; Zhang H. J. et al., 2021). When an extracellular signal activates MAPK on the cell membrane, MAPK3 phosphorylates and activates MAPK2, after which MAPK2 phosphorylates the threonine/tyrosine residues of MAPK. This causes MAPK to be activated and translocated to interact with transcription factors or downstream kinases in the nucleus, thus affecting the transcription of target genes and regulating the inflammatory response. Currently, p38 MAPK, JNK, and ERK1/2 have been extensively studied. They are some of the common crossover pathways of signal transduction, such as inflammation, cell proliferation, differentiation, apoptosis, and oxidative stress, and are closely related to DR (Papa et al., 2019; Hammouda et al., 2020; Li X. H. et al., 2022; Zhou et al., 2022).

Curcumolide (Cur) is a sesquiterpenoid compound extracted from *Curcuma wenyujin* Y.H.Chen et C. Ling, a TCM for promoting blood circulation and removing blood stasis. Cai Y. et al. (2017) found that Cur reduced the inflammatory damage to retinal vascular endothelium and decreased the expression of TNF-α and ICAM-1 in diabetic rats. Further *in vitro* experiments were performed to explore the molecular mechanisms of Cur. The results showed that Cur significantly inhibited the p65 nuclear translocation of human umbilical vein endothelial cells (HUVECs) induced by TNF-α and inhibited p-p38, p^ser536^-p65, p^ser276^-p65, p-IKKβ, and p-IκBα, which showed a dose-dependent relationship. In addition, PCR results showed that Cur downregulated the mRNA expression of pro-inflammatory mediators TNF-α and ICAM-1. These results suggested that Cur may inhibit the p38/NF-κB signaling pathway to regulate the expression of pro-inflammatory mediators, thereby alleviating retinal inflammation in DR. Zhang et al. (2019) isolated the benzyl compound erianin from *Dendrobium chrysotoxum* Lindl. (DC) and inhibited the expression of TNF-α, p-ERK1/2, p-cRaf, p-MEK1/2, p-p65, p-IκB, and p-IKK *in vivo* and *in vitro*. It was also able to upregulate claudin-1 and occludin levels and alleviate BRB breakdown in the retinas of diabetic mice. It has been suggested that erianin improves retinal inflammation by inhibiting the activation of the ERK1/2-NF-κB signaling pathway, thereby alleviating BRB breakdown during DR.

In addition, the TCM formulas LuoTong formula (LTF) (Pang et al., 2020a) and Hu-Zhang-Qing-Mai-Yin (Yu et al., 2021) ameliorated DR by inhibiting the activation of the p38 MAPK/NF-κB signaling pathway.

### 2.3 TLR4/NF-κB signaling pathway

Toll-like receptor 4 (TLR4), also known as CD284, is a member of the TLR family. TLR4 is highly expressed in diabetic patients and can promote an inflammatory response. TLR4 activates NF-κB through MyD88 and TRIF pathways. The cascade stimulation of TLR4 and NF-κB signals can aggravate the inflammatory response caused by diabetes, thus promoting the development of DR (Li, X. H. et al., 2022; Wen et al., 2020; Shu et al., 2021). Nimbolide is a terpenoid found mainly in the neem plant parts. Shu et al. (2021) reported the intervention effect of nimbolide on DR. They found that nimbolide reduced the levels of the pro-inflammatory factors TNF-α, IL-1β, and IL-6 in the retinal tissue of diabetic rats, as well as the levels of MCP-1, MMP-9, and VEGF in the serum. In addition, nimbolide reduced the mRNA levels of TLR4 and NF-κB in the retinal tissue of diabetic rats and improved the retinal thickness and cell number. Nimbolide has been suggested to improve DR by inhibiting the TLR4/NF-κB signaling pathway. Moreover, Pan et al. (2021) demonstrated that the Qi-Ming granule improves retinal inflammation in diabetic rats, and its mechanism may be related to the inhibition of the HMGB1/TLR4/NF-κB signaling pathway.

### 2.4 Other signaling pathways

Quercetin is a natural flavonoid that is widely found in the plant kingdom and has anti-inflammatory and antioxidant properties (Shen et al., 2021). Chai et al. (2021) reported an intervention effect of quercetin on STZ-induced diabetic rats. Quercetin significantly increased the number of ganglion cells and the thickness of the retinal cell layer. Moreover, quercetin decreased the expression of IL-1β, IL-6, IL-18, TNF-α, high mobility group box-1 (HMGB1), NLRP3, ASC, caspase-1, TLR4, NF-κBp65, VEGF, and sICAM-1, and increased the expression of HO-1, BDNF, and NGF in the retinal tissue of diabetic rats. It has been suggested that quercetin is beneficial to DR through its anti-inflammatory, anti-angiogenic, and neurotropic effects, which are related to the upregulation of HO-1 and inhibition of the HMGB1/TLR4/NF-κB/NLRP3 inflammasome/IL-1β/IL-18 axis. Ran et al. (2019) found that curcumin decreased the expression of inflammatory cytokines IL-1β, IL-6, and TNF-α and downregulated the formation of ROS and the levels of p-AKT and p-mTOR in HG-induced retinal pigment epithelial cells (RPECs). These results suggest that curcumin inhibits inflammation in DR via a mechanism related to the ROS/PI3K/AKT/mTOR signaling pathway.

## 3 Signaling pathways in TCM intervening with angiogenesis of DR

**FIGURE 3:**
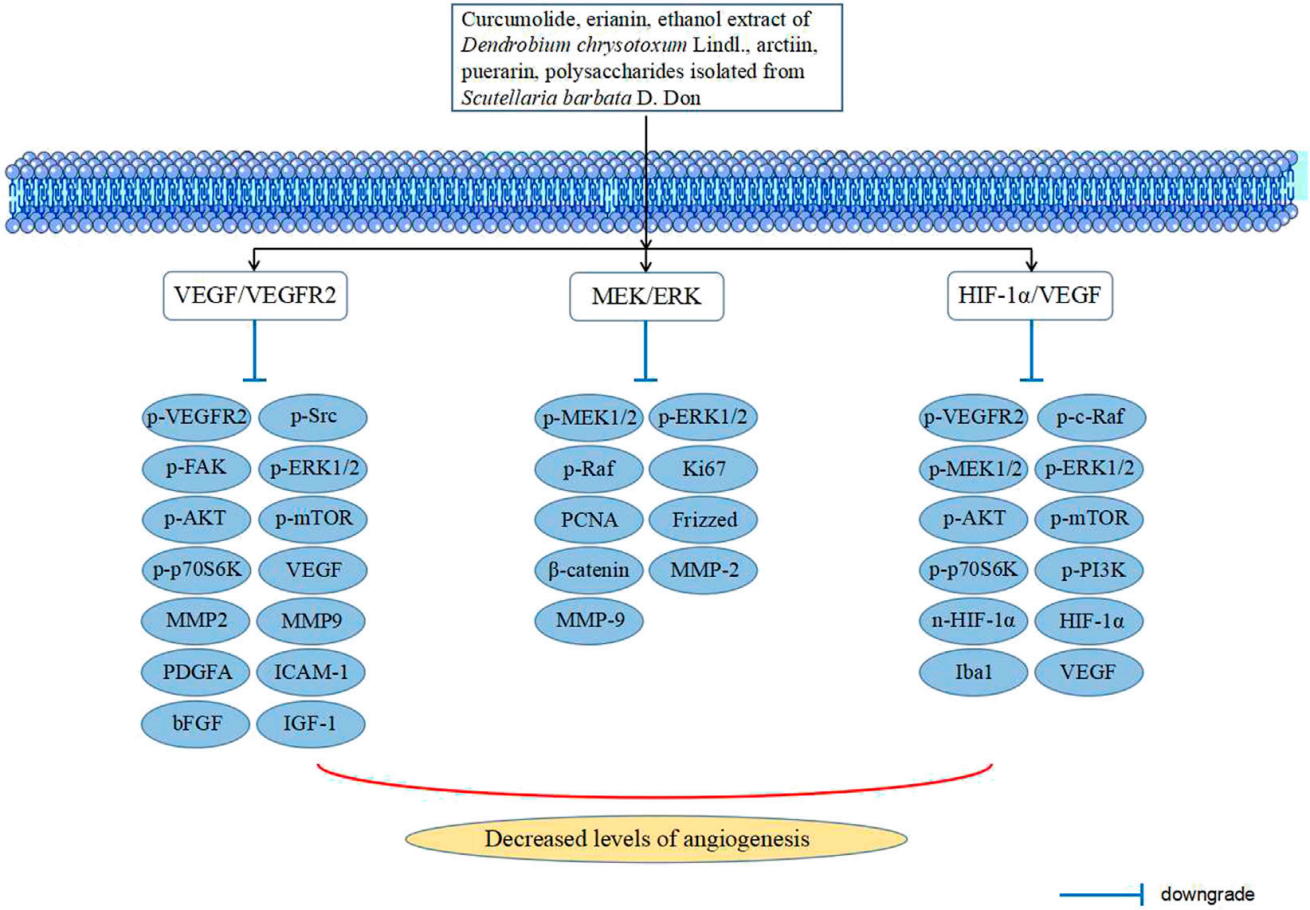
Signaling pathways in TCM intervening with angiogenesis of DR.

It is well known that PDR belongs to the middle and later stages of DR. The most significant difference between PDR and NPDR is neovascularization due to retinal ischemia and hypoxia and vascular leakage, which is a rapid and uncontrolled process of retinal angiogenesis. Neovascularization is fragile and prone to rupture and bleeding, eventually causing irreversible retinal damage and blindness (Yu et al., 2016a; Lin et al., 2019). Furthermore, retinal ischemia induces the production of angiogenic factors in the vitreous cavity, leading to retinal vascular proliferation. Hypoxia is closely related to the expression of HIF-1α, and they are positively related to the expression of vascular endothelial growth factor (VEGF) and neovascularization (Ai et al., 2020). In recent years, many researchers have investigated the effect of TCM on DR angiogenesis (Figure 3), and the signaling pathways involved are explained in the following paragraphs.

### 3.1 VEGF/VEGFR2 signaling pathway

VEGF, a glycoprotein in the growth factor family, is the most important cytokine in angiogenesis and is closely related to retinal vascular permeability. The upregulated expression of VEGF and its abnormal signaling can stimulate pathological angiogenesis, including neovascularization in the middle and late stages of DR. The tyrosine kinase receptors of VEGF are highly specific transmembrane receptors, including VEGFR1, VEGFR2, VEGFR3, neuropilin-1, and neuropilin-2, which bind to VEGF and activate the intracellular signaling cascade through autophosphorylation. This signaling is then progressively transduced and amplified, which disrupts the expression of relevant pro-angiogenic factors and activates downstream signaling pathways, leading to the proliferation and migration of retinal vascular endothelial cells, increased vascular permeability, and substantial angiogenesis. Among them, VEGFR2 is mainly expressed and produced in vascular endothelial cells, which leads to pathological angiogenesis and plays an important role in the development of PDR (Yu et al., 2017; Lin et al., 2019; Kaštelan et al., 2020; Eguchi et al., 2022).

Cur has been mentioned previously to reduce retinal inflammation in diabetes. However, in addition to its anti-inflammatory effects, Cur may improve DR via anti-angiogenesis. Lin et al. (2019) found that Cur significantly inhibited the avascular area and the number of neovascular lumens and neovascular clusters in the retina of oxygen-induced retinopathy (OIR) mice. *In vitro* experiments showed that Cur inhibited the migration, apoptosis, tube formation, and proliferation of VEGF-induced HUVECs. These experimental results indicate the antiangiogenic ability of Cur. In addition, molecular mechanism studies showed that Cur not only inhibited the tyrosine kinase activation of VEGFR2 by down-regulating the phosphorylation of VEGFR2 but also inhibited the activation of VEGFR2-mediated downstream signaling pathways, such as c-Src, FAK, ERK1/2, AKT, mTOR, and p70S6K. These results suggested that Cur may be a potential angiogenic antagonist that plays a significant anti-angiogenic role in the treatment of DR by inhibiting the VEGF/VEGFR2 signaling pathway, especially neovascularization in PDR.

Gong et al. (2014) found that after the ethanol extract of DC intervention, retinal vascular density was decreased in diabetic rats. DC also inhibited the levels of VEGF and VEGFR2 in the retinal tissue of diabetic rats, and matrix metalloproteinase2/9 (MMP2/9), basic fibroblast growth factor (bFGF), platelet-derived growth factor A (PDGFA), and insulin-like growth factor 1 (IGF-1) in the serum, which are closely related to promoting angiogenesis. Similar to Cur, the ethanol extract of DC not only reflects its role in anti-angiogenesis but also plays a remarkable role in retinal inflammation, which is related to its inhibition of proinflammatory factors and phosphorylation of p65. In addition, LTF could improve DR by reducing the level of VEGF/VEGFR and increasing the level of PEDF to repair blood vessels (Pang et al., 2020b).

### 3.2 HIF-1α/VEGF signaling pathway

Hypoxia-inducible factor-1α (HIF-1α) is a highly conserved transcription factor prevalent in mammals under anoxic conditions that consists of the *ß*-subunit that constitutes its expression and the α-subunit that regulates oxygen. It is difficult for it to exist in a normoxic environment, and it rapidly degrades. However, once it enters an anoxic environment, it stabilizes and then migrates into the nucleus and combines with anoxic response elements on the VEGF promoter to upregulate the expression of VEGF (Teng et al., 2009; Chen et al., 2019; Li X. H. et al., 2022).

Erianin has both anti-inflammatory and anti-angiogenic abilities, the anti-inflammatory abilities of which have been described previously. Yu et al. (2016a) revealed the anti-angiogenic mechanism of erianin. *In vivo* experiments showed that erianin alleviated retinal neovascularization in STZ-induced and OIR mice. Moreover, erianin downregulated the nuclear expression of VEGF, p-VEGFR2, and HIF-1α *in vitro*. It also inhibits the expression of p-c-Raf, p-MEK1/2, p-ERK1/2, p-PI3K, p-Akt, p-mTOR, and p-P70S6Kinase downstream of VEGFR2. These results suggested that erianin exerts its anti-angiogenic effects by blocking the HIF-1α/VEGF/VEGFR2 signaling pathway and its downstream signaling pathway. Furthermore, Yu et al. (2016b) found in another study that the ethanol extract of DC improves DR angiogenesis by inhibiting the HIF-1α/VEGF/VEGFR2 signaling pathway.

Both erianin and the ethanol extract of DC have shown strong potential against DR in terms of anti-inflammatory and anti-angiogenesis, which also suggests that researchers can strengthen research on DC and provide a more experimental basis for the clinical application of DC in the treatment of DR. In addition, puerarin (Teng et al., 2009) and resveratrol (Liu et al., 2021) regulate the HIF-1α/VEGF signaling pathway, and arctiin (Fu et al., 2016) regulates the AKT/HIF-1α/VEGF-A signaling pathway, both of which improve DR to varying degrees.

### 3.3 Other signaling pathways

*Scutellaria barbata* D. Don (SB) has a wide range of biological activities including antitumor and anti-inflammatory properties (Chen et al., 2020). In a previous study (Mei et al., 2017), the ethanol extract of SB was shown to improve DR by reducing retinal inflammation via the NF-κB signaling pathway. Additionally, SB may improve DR in other ways. Li and Xiao (2021) found that polysaccharides isolated from SB (PSB) inhibited high glucose (HG)-induced human retinal vascular endothelial cell (HRVEC) proliferation, migration, and neovascularization by regulating the activation of the MEK/ERK signaling pathway and the VEGF/VE-cadherin axis. Radix Trichosanthis (RT) is often used to treat diabetes, and ethyl acetate extracts of RT (ERT) have been found to significantly inhibit HG-induced retinal vascular endothelial cell (RVEC) proliferation, migration, and tube formation, which are related to the regulation of the Hippo and Notch signaling pathways (Song et al., 2019). Fufang Xueshuantong is composed of *Panax notoginseng* (Burk) F. H. Chen, *Salvia miltiorrhiza* Bunge, *Astragalus propinquus* Schischkin, and *Scrophularia ningpoensis* Hemsl., which showed effects similar to ERT in RVECs and decreased the expression of VEGF, which were proven to be through targeting YAP-mediated pathways (Xing et al., 2019). The aforementioned TCM all show prospects against DR, especially PDR.

## 4 Signaling pathways in TCM intervening with oxidative stress of DR

**FIGURE 4:**
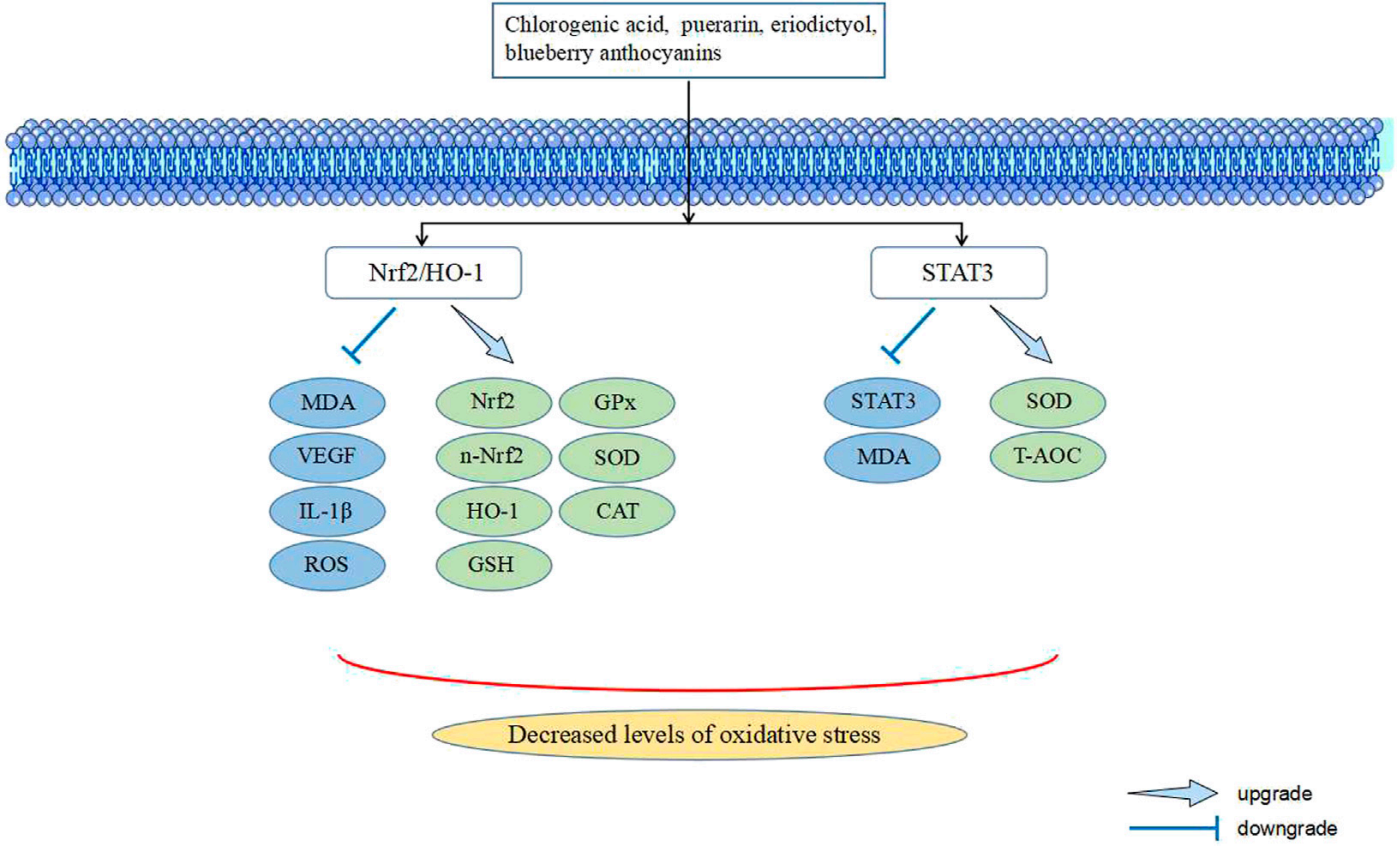
Signaling pathways in TCM intervening with oxidative stress of DR.

A series of metabolic abnormalities caused by persistent hyperglycemia, such as the formation of advanced glycation end products (AGEs), polyol pathway, protein kinase C (PKC) pathway, and hexosamine pathway, leads to an excessive concentration of reactive oxygen species (ROS) and even the destruction of the dynamic oxygen reduction balance when the antioxidant capacity is exceeded, which causes oxidative stress. The retina is relatively susceptible to oxidative stress because it is a high oxygen-consuming organ, owing to its visual imaging function and active metabolic function, which is conducive to the formation of ROS under high oxygen tension. In addition, oxidative stress can cause inflammation, mitochondrial dysfunction, lipid peroxidation, apoptosis, and retinal structure and function damage, ultimately leading to DR. Oxidative stress has been proven to be one of the pathological mechanisms of DR. In recent years, the relevant signaling pathways related to TCM against DR have been described (Figure 4) (Ola et al., 2018; Cecilia et al., 2019; Kang and Yang, 2020).

### 4.1 Nrf2/HO-1 signaling pathway

Nuclear factor erythroid 2-related factor 2 (Nrf2) is a key transcription factor that maintains cellular redox homeostasis. Under normal oxygen conditions, Nrf2 is mainly distributed in the cytoplasm and forms complexes with the link protein Kelch-like ECH-associated protein 1 (KEAP1). KEAP1 mediates the ubiquitination of Nrf2 and is a negative regulator of Nrf2. Under oxidative stress, with an increase in ROS production, Nrf2 is released from the complex and enters the nucleus, where it combines with the promoter antioxidant response element (ARE) sequence to induce downstream gene transcription, such as heme oxygenase-1 (HO-1), glutathione peroxidase (GPx), superoxide dismutase (SOD), NADPH quinone oxidoreductase 1 (NQO1), and catalase (CAT) (Li B. et al., 2020; Ulasov et al., 2022; Yuan et al., 2022). Among them, HO-1 is an important stress-induced reactive protein, one of the phase II antioxidant enzymes regulated by Nrf2, which can catalyze the conversion of hemoglobin into CO, Fe^2+^, and biliverdin, and finally convert the aforementioned substances into bilirubin, thus playing a role in antioxidant stress (Zhang Q. et al., 2021; Chiang et al., 2021).

Blueberry is rich in antioxidants, which protect against many chronic diseases, and is one of the foods with the highest levels of anthocyanins (Felgus-Lavefve et al., 2022). Song et al. found that blueberry anthocyanins (BA) reversed weight loss and increased blood glucose in diabetic rats, and also upregulated the antioxidant capacity of the retina by increasing the activity of glutathione (GSH) and GPx and reducing the level of malondialdehyde (MDA) and ROS. In addition, BA upregulated the mRNA levels of Nrf2 and HO-1, as well as the protein levels of nuclear-Nrf2 and HO-1. These results suggested that BA may protect the retina from diabetes-induced oxidative stress by regulating the Nrf2/HO-1 signaling pathway (Song et al., 2016). Lv et al. (2019) found that eriodictyol reduced ROS production and increased the activities of SOD, GPx-1, and CAT in HG-induced rat retinal ganglion cells (RGCs), which reflects the antioxidant stress ability of eriodictyol. Eriodictyol also increased the nuclear translocation of Nrf2 and expression of HO-1, suggesting that the antioxidant stress ability of eriodictyol may be realized by regulating the Nrf2/HO-1 signaling pathway. Moreover, Ouyang et al. (2022) found that chlorogenic acid improved TNF-α induced oxidative injury in human retinal endothelial cells (HRECs) by inducing Nrf2 activation.

### 4.2 STAT3 signaling pathway

STAT3 is a redox regulatory protein. Its biological function is affected by the cellular redox environment and is mainly involved in cell proliferation, survival, apoptosis, immune response, and tumor occurrence and development. In its resting state, STAT3 exists in the cytoplasm as a monomer or non-phosphorylated dimer. When stimulated by ROS, STAT3 is activated through the phosphorylation of tyrosine 705, which leads to its translocation into the nucleus and transcription (Xu et al., 2019; Butturini et al., 2020; Tošić and Frank, 2021).

*Pueraria lobata* (Willd.) Ohwi is a popular natural antioxidant in TCM with medicinal and food homologs. Puerarin is the main active ingredient of *P. lobata* (Willd.) Ohwi, which has antioxidant, anti-inflammatory, and antitumor activities, and has a positive effect on diabetes and its complications (Bai et al., 2022; Wang et al., 2022). Cai Y. H. et al. (2017) reported the effect of puerarin on retinal oxidative stress in diabetic rats induced by STZ. Puerarin increased the activity of SOD in the serum and retina, increased the T-AOC content in the retina, and decreased the MDA content in the serum and retina, reflecting the antioxidant capacity of puerarin. In addition, puerarin reduced blood glucose and insulin levels in diabetic rats and significantly inhibited mRNA and protein expression of STAT3. These results suggested that the ability of puerarin to reduce retinal oxidative stress damage is related to the inhibition of STAT3 expression.

## 5 Signaling pathways in TCM intervening with other pathological progressions of DR

Many TCM improve DR through inflammation, angiogenesis, and oxidative stress, but a few TCM improve DR through other processes, such as apoptosis and autophagy. Furthermore, some studies on TCM against DR have not explicitly stated which process is used to improve DR.

*Cyclocarya paliurus* (Batalin) Iljinsk. (CP) is a TCM for both medicine and food, which promotes the secretion of saliva or body fluid and clears away heat and toxic material. Arjunolic acid (AA) is an oleanane triterpenoid extracted from CP, and Zhang et al. (2022) found that the expression of Bcl-2/Bax and caspase-3 decreased in STZ-induced diabetic rats during AA intervention, reflecting the anti-apoptotic effect of AA. In addition, AA downregulated the levels of TNF-α, IL-6, and IL-1β and upregulated the levels of HO-1 in retinal tissue, suggesting the antioxidant and anti-inflammatory potential of AA. These effects of AA were confirmed again in H_2_O_2_-induced ARPE-19 cells. Further mechanistic studies showed that AA upregulated the levels of LC3II/I and p-AMPK, and downregulated the levels of p62 and p-mTOR both *in vivo* and *in vitro*. These results indicated that AA improved apoptosis, oxidative stress, and inflammation in retinal cells through the AMPK/mTOR/HO-1 autophagy signaling pathway. Shabyar (SBA) is a TCM formula that can be used to treat vision loss caused by DR. After SBA intervention in the HG-induced APRE-19 model, SBA reduced the levels of AR, ROS, sorbitol, Beclin-1, Atg3, Atg5, Atg7, LC3 II, and Bax/Bcl2 ratio. The expression of pAMPK/AMPK and pULK1/ULK1, which are associated with autophagy initiation, was reversed. In addition, SBA increased pmTOR/mTOR, SQSTM1/p62, and MMP levels, alleviated cell edema, and reduced intracellular autophagosomes. These results suggested that SBA inhibited autophagy and early apoptosis of ARPE-19 cells via the AMPK/mTOR/ULK1 signaling pathway (Liu X. Y. et al., 2022). Bie-Jia-Ruan-Mai-Tang (BJ) is a TCM formula used to treat PDR. BJ inhibited acellular capillary formation and VEGF expression in the retina of diabetic mice. Moreover, BJ increased ROS production, decreased ATP production and mitochondrial membrane potential, inhibited cell proliferation, and induced apoptosis in HG-induced HRCECs. The effects of BJ on DR are related to the regulation of the PI3K/AKT and NF-κB signaling pathways (Liu et al., 2022b). Xiao Bopi (XBP), derived from the classic Tibetan medicine *Berberis dictyophylla* F., has the effect of clearing heat and decreasing mKhris-pa and can be used clinically to treat diabetes and its complications. XBP inhibited the HIF-1α/VEGF/DLL-4/Notch-1 signaling pathway to reduce angiogenesis and apoptosis, thus improving DR (Ai et al., 2022). Additionally, berberine (Wang et al., 2021) regulated the TLR4/STAT3/VEGF signaling pathway, and Fufang Xueshuantong (Sun et al., 2021) regulated the PPAR signaling pathway and complement and coagulation cascades to improve DR.

## 6 Discussion

TCM is the crystallization of wisdom summed up after thousands of years of clinical practice and has played an important role in the history of the healthy development of Chinese people. In the past, TCM was used to treat various diseases. They considered whether the symptoms of the disease improved after taking the drug but did not care about the way TCM played its role. At that time, the technology was insufficient to reveal the mechanism of action of TCM. As science and technology have advanced, researchers have gradually uncovered the laws of the signaling pathways that mediate life activity between cells. When stimulated by an external signal, cells initiate an intracellular signaling cascade that transforms the extracellular signal into an intracellular signal, which exerts biological effects through signal amplification, modification, and regulation. In addition, with the growing popularity of TCM worldwide, there has been a growing interest in the identification of bioactive compounds, and almost half of all small-molecule drugs developed in the last dozen years have been related to natural products, of which TCM is an essential component. Correspondingly, based on these bioactive compounds, with further structural modifications, these compounds are considered invaluable sources of potential therapeutic drugs, demonstrating the importance of identifying bioactive compounds. According to previous research conclusions (Li W. C. et al., 2022), this review innovatively considers the core pathological mechanisms in the development processes of DR (such as inflammation, angiogenesis, and oxidative stress) as the fundamental unit and expounds on the mechanism and potential of TCM against DR from the perspective of signaling pathways (Table 1). Inflammation plays a dominant role in the early stage of DR. TCM can improve inflammation through the NF-κB, MAPK/NF-κB, and TLR4/NF-κB signaling pathways against DR. Coincidentally, all these mechanisms are related to the NF-κB signaling pathway, suggesting that this pathway is a potential target for diabetic retinal inflammation and an important entry point for delaying the progression of DR pathology. More attention should be paid to the NF-κB signaling pathway, and on this basis, its synergies with other related signaling pathways in DR can be explored, which may be a more promising approach against DR. Angiogenesis is another important pathological mechanism in the development of DR, and it is present throughout almost the entire course of PDR. TCM inhibited angiogenesis through the VEGF/VEGFR2, HIF-1α/VEGF, MEK/ERK, Hippo, Notch, and YAP-mediated signaling pathways against DR. Among these, VEGF is the most important cytokine in angiogenesis, and the results of this study demonstrate the importance of VEGF-related signaling pathways in angiogenesis. An in-depth study of VEGF-related signaling pathways is an effective way to inhibit angiogenesis. At present, there are quite a few studies on TCM intervention for oxidative stress and apoptosis in DR, but the contents of these studies tend to explain the antioxidant and anti-apoptotic effects of TCM rather than the signaling pathways. Therefore, few related studies have been included, and the signaling pathways involved mainly include Nrf2/HO-1, STAT3, AMPK/mTOR/HO-1, AMPK/mTOR/ULK1, HIF-1α/VEGF/DLL-4/Notch-1, and PI3K/AKT. These results indicate that many TCMs can inhibit the inflammatory, angiogenic, oxidative stress, and apoptosis processes of DR by regulating crosstalk among signaling pathways through multi-target synergy. In addition, some TCMs can be used against DR by intervening in multiple pathological processes, such as erianin inhibition of retinal inflammation and angiogenesis through the ERK1/2-NF-κB and HIF-1α/VEGF/VEGFR2 signaling pathways. Similarly, Cur can inhibit retinal inflammation through the p38 MAPK/NF-κB signaling pathway and inhibit angiogenesis through the VEGF/VEGFR2 signaling pathway, thus exerting a beneficial influence on DR. Considering the complexity of the pathological mechanism of DR, TCM with multiple pathways and layers has broad prospects as a candidate drug for the treatment of DR. Although TCM has shown great promise in the treatment of DR, it still has some limitations. First, the different origins may lead to different components of TCM, affecting its efficacy and safety. Second, there is a lack of large-scale clinical trials on TCM treatments for DR. Third, the experimental design of most TCM formulas and extracts for DR treatment lacks strict positive and negative controls. Finally, in recent years, studies on the improvement of DR through the intervention of TCM in the autophagy process have gradually emerged, but the relevant autophagy signaling pathways have rarely been explored. Therefore, investigating whether TCM can inhibit DR by regulating autophagy-related signaling pathways may be a direction that needs further exploration.

**TABLE 1:** *In vivo* and *in vitro* experimental evidence of TCM in the treatment of DR.

Pathological progression	Agent	Active compounds and plants	Experiment model		Molecular mechanism	Signaling pathway	Reference
			*In vivo*	*In vitro*			
Inflammation	Curcumolide (Cur)	*Curcuma wenyujin* Y.H.Chen et C.Ling	Wistar rats	HUVECs	↓: TNF-α, ICAM-1, p-IKKβ, p-IκBα, p^ser536^-p65, p^ser276^-p65, nucleus-p65, and p-p38	p38MAPK/NF-κB	Cai Y. et al. (2017)
Inflammation	Ethanol extract of *Scutellaria barbata* D. Don	*Scutellaria barbata* D. Don	C57BL/6J mice	-	↓: TNF-α, IL-1β, ICAM-1, p-p65, nucleus-p65, and Iba1	NF-κB	Mei et al. (2017)
					↑: claudin-1 and claudin-19		
Inflammation	Erianin	*Dendrobium chrysotoxum* Lindl.	C57BL/6 mice	BV2 cells	↓: TNF-α, p-ERK1/2, p-cRaf, p-MEK1/2, p-IκB, p-p65, nucleus-p65, and p-IKK	ERK1/2-NF-κB	Zhang et al. (2019)
					↑: claudin1 and occludin		
Inflammation	Shengpuhuang-tang (ST)	*Typha angustifolia* L., *Eclipta prostrata* L., *Nelumbo nucifera* Gaerth., *Salvia miltiorrhiza* Bge., *Paeonia suffruticosa* Andrews, *Rehmannia glutinosa* Libosch, *Curcuma wenyujin* Y. H. Chen et C. Ling, *charred Schizonepeta tenuifolia* Briq., *Gardenia jasminoides* Ellis, *Ligusticum chuanxiong* Hort., and *Glycyrrhiza uralensis* Fisch	Wistar rats	-	↓: TNF-α, ICAM-1, p-p65, p-IKKβ, and nucleus-p65	NF-κB	Li W. C. et al. (2020)
					↑: IκBα		
Inflammation	LuoTong formula (LTF)	*Astragalus membranaceus* (Fisch.) Bunge, *Salvia miltiorrhiza* Bunge, *Panax notoginseng* (Burk) F.H. Chen, *Hirudo nipponica* Whitman, and *Rheum palmatum* L	SD rats	-	↓: TNF-α, ICAM-1, p-p38, IL-1β, MCP-1, and nuclear-NF-κBp65	p38MAPK/NF-κB	Pang et al. (2020a)
Inflammation	*Celosia argentea* L. extract (CAE)	*Celosia argentea* L	C57 mice	-	↓: TNF-α, IL-1β, IL-6, VEGF, p-NF-κB, and p-IKK	NF-κB	Guo et al. (2016)
					↑: IκB		
Inflammation	Curcumin	-	-	RPECs	↓: IL-1β, IL-6, TNF-α, ROS, p-AKT, and p-mTOR	ROS/PI3K/AKT/mTOR	Ran et al. (2019)
Inflammation and angiogenesis	Quercetin	-	SD rats	-	↓: IL-1β, IL-6, IL-18, TNF-α, HMGB1, NLRP3, ASC, caspase-1, TLR4, NF-κBp65, sICAM-1, and VEGF	HMGB1/TLR4/NF-κB/NLRP3	Chai et al. (2021)
					↑: HO-1, BDNF, and NGF		
Angiogenesis	Curcumolide (Cur)	*Curcuma wenyujin* Y.H. Chen et C. Ling	C57BL/6J mice	HUVECs	↓: p-VEGFR2, p-Src, p-FAK, p-ERK1/2, p-AKT, p-mTOR, and p-p70S6K	VEGF/VEGFR2	Lin et al. (2019)
Angiogenesis	Erianin	*Dendrobium chrysotoxum* Lindl.	C57BL/6 mice	RF/6A cells, BV2 cells	↓: VEGF, p-VEGFR2, Iba1, nucleus-HIF-1α, p-ERK1/2, p-c-Raf, p-MEK1/2, p-PI3K, p-AKT, p-mTOR, and p-p70S6K	HIF-1α/VEGF/VEGFR2	Yu et al. (2016a)
Angiogenesis	Ethanol extract of *Dendrobium chrysotoxum* Lindl. (DC)	Erianin, moscatilin, and gigantol, and *Dendrobium chrysotoxum* Lindl.	SD rats	-	↓: VEGF, VEGFR2, MMP2, MMP9, PDGFA, bFGF, IGF-1, ICAM-1, IL-6, IL-1β, and p-p65	VEGF/VEGFR2	Gong et al. (2014)
Angiogenesis	Polysaccharides isolated from *Scutellaria barbata* D. Don	*Scutellaria barbata* D. Don	-	HRVECs	↓: PCNA, Ki67, p-Raf, p-ERK1/2, p-MEK1/2, VE-cadherin, frizzed, *ß*-catenin, MMP-2, and MMP-9	MEK/ERK	Li and Xiao (2021)
Angiogenesis	Bie-Jia-Ruan-Mai-Tang (BJ)	*Trionyx sinensis* Wiegmann, *Acorus tatarinowii*, *Sedum sarmentosum*, and *Paeonia lactiflora*	C57BL/6J mice	HRCECs	↓: VEGF, p-PI3K, P-AKT, and Bcl-xL	PI3K/AKT, NF-κB	Liu et al. (2022b)
					↑: ZO-1, ROS, Bax, and p-NF-κB		
Oxidative stress	Blueberry anthocyanins (BA)	-	SD rats	-	↓: MDA, ROS, VEGF, and IL-1β	Nrf2/HO-1	Song et al. (2016)
					↑: GSH, GPx, HO-1, Nrf2, and nucleus-Nrf2		
Oxidative stress	Eriodictyol	-	-	RGCs	↓: ROS	Nrf2/HO-1	Lv et al. (2019)
					↑: SOD, GPx-1, CAT, Nrf2, and HO-1		
Oxidative stress	Puerarin	*Pueraria lobata* (Willd.) Ohwi	SD rats	-	↓: MDA and STAT3	STAT3	Cai Y. H. et al. (2017)
					↑: SOD and T-AOC		
Apoptosis, oxidative stress, and inflammation	Arjunolic acid	*Cyclocarya paliurus* (Batalin) Iljinsk	SD rats	-	↓: Bax/Bcl-2, caspase-3, IL-1β, IL-6, TNF-α, p62, and p-mTOR	AMPK/mTOR/HO-1	Zhang et al. (2022)
					↑: LC3II/I, HO-1, and p-AMPK		
Apoptosis and autophagy	Shabyar	*Aloe vera* (L.) Burm.f., *Rosa rugosa* Thunb., *Operculina turpethum* (L.) Silva Manso, *Pistacia lentiscus* L., *Terminalia chebula* Retz., *Foeniculum vulgare* Mill., and *Tribulus terrestris* L	-	APRE-19	↓: AR, ROS, sorbitol, Beclin-1, Atg3, Atg5, Atg7, LC3II, Bax/Bcl2 ratio, pAMPK/AMPK, and pULK1/ULK1	AMPK/mTOR/ULK1	Liu X. Y. et al. (2022)
					↑: pmTOR/mTOR, SQSTM1/p62, and MMP		
Apoptosis and angiogenesis	Water extract of Xiao Bopi (XBP)	Magnoflorine, jatrorrhizine, palmatine, and berberine, *Berberis dictyophylla* F	db/db mice	-	↓: HIF-1α, VEGF, DLL-4, Notch-1, Bax, Apaf-1, Cyto-c, cleaved Caspase-3, and cleaved caspase 9	HIF-1α/VEGF/DLL-4/Notch-1	Ai et al. (2022)
					↑: Bcl-2		

↑, upgrade; ↓, downgrade

In summary, TCM should be used to carry out in-depth research on the prevention and treatment of DR in the future, explore the pathogenesis of DR, and identify potential targets for screening candidate drugs against DR to provide ideas for the development of new drugs against DR.
